# Role of Self-Control and Self-Construal in the Army Morale and Suicidal Ideation of Chinese Military Cadets

**DOI:** 10.3389/fpsyg.2022.904170

**Published:** 2022-06-02

**Authors:** Outong Chen, Ranran Liu, Xiaojing Zhao

**Affiliations:** ^1^Department of Psychology, Normal College and School of Teacher Education, Qingdao University, Qingdao, China; ^2^Qingdao Branch, Naval Aeronautical University, Qingdao, China; ^3^Weifang Engineering Vocational College, Qingzhou, China

**Keywords:** military cadet, army morale, suicidal ideation, self-control, self-construal

## Abstract

The current study investigated the relationship between army morale and suicidal ideation in Chinese military cadets, including the mediating role of self-control and the moderating role of self-construal. A total of 1124 male navy cadets participated in the study, completing a series of questionnaires. The results revealed the following: (1) army morale could negatively predict suicidal ideation; (2) the negative predictive effect of army morale on suicidal ideation could be partially mediated by self-control; and (3) self-construal moderated the predictive effect of army morale on suicidal ideation among navy cadets. Finally, the current study suggested that building some relevant assessment, diagnostic, and training programs may help build army morale and further prevent suicidal ideation in the military context.

## Introduction

According to the World Health Organization (WHO), suicide is becoming a leading cause of death worldwide. As of 2019, the number of suicide cases exceeded the number of deaths due to war, homicide, HIV, and breast cancer.^1^ Among the different occupations, soldiers are a high-risk population for suicide. As far back as 2008, the suicide rate in the United States military was already higher than the adjusted civilian rate, reaching its highest point in nearly three decades (Bush et al., 2013). Moreover, this rate was still increasing till 2020.^2^

In contrast to the general population, soldiers face unique problems that may lead to suicide. A study suggested that soldiers’ suicidal behavior may occur before, during, or after a military deployment or service (Martin et al., 2009). Multiple issues associated with soldiers’ suicides remain unexplained despite previous existent research. Based on scale measurements, the current study investigated if military cadet morale impacted suicidal ideation.

### Army Morale and Suicidal Ideation

Previous studies defined morale as the capacity of a specific group of people to become united as one, persistently and consistently, in pursuit of a common goal (Leighton, 1949). Key to morale is an individual’s ability to maintain faith, especially in the face of opposition and hardship. Morale is often considered to be correlated with specific psychological traits, such as self-discipline (Akhtar et al., 2016) and willpower (Chinyere et al., 2020), among others. It also has an impact on the group level. Olivetta’s study suggested that morale could promote group cohesion and operational effectiveness (Olivetta, 2017); it plays an important role in modern military effectiveness (Bowers et al., 1994) and combat performance (Fennell, 2014). Based on multiple studies, Li concluded that army morale can be understood from two different perspectives: personal and group (Li, 2006). The group perspective can be summarized as the cohesion or identification of a soldier with a military unit. The higher the alignment of a soldier’s individual goals with the unit’s goals, the higher the soldier’s identification will be with the military unit. If a force has a positive morale, it may be less likely to surrender during combat. From a personal perspective, army morale is related to many psychological traits, such as self-discipline and willpower. Some researchers have indicated that it is also related to an individual’s well-being and adjustment (Fennell, 2014); they have proposed that a soldier’s morale is a state in which the needs of the soldier are met adequately or are likely to be met.

Suicidal ideation is defined as the intention to harm oneself without taking actions that threaten one’s own survival (Lin et al., 2018). In contrast to suicidal behaviors, individuals generally express suicidal ideation before committing to suicidal actions (Jiang, 2015). Thus, suicidal ideation can be considered a leading predictor of suicidal behavior. Some evidence suggests that higher life satisfaction and happiness could have significant preventive effects on suicidal ideation among college freshmen (Liu et al., 2012). Jin and Jie also stated that well-being could prevent suicidal ideation (Jin and Zhang, 1998). Moreover, psychological well-being may be a much stronger predictor of suicidal ideation than physical well-being. From the personal perspective, existent research opines that psychological traits, including well-being (Fennell, 2014) and willpower (Chinyere et al., 2020), are central to army morale. These previous studies have indirectly indicated that there may be a potential relationship between morale and suicidal ideation in a military context.

Moreover, army morale may also be linked with suicidal ideation from a group perspective. Joiner et al. (2009) propounded the interpersonal-psychological theory of suicide and suggested that a lack of social belonging and the existence of social alienation are key to suicidal ideation. Consistent with the group perspective on army morale, this view involves many social characteristics, including group identification, alignment, and cohesiveness (Li, 2006). Jones et al. (2012) suggested that individuals in military units with higher morale may receive both physical and emotional support from leaders and peers. Such an atmosphere is beneficial for individuals’ mental health. This interpretation is supported by Sipos et al. (2017). Their study revealed a significant negative correlation between morale and factors that increase risks to mental health, such as stress, depression, and anxiety. Furthermore, other researchers believe morale is a typical positive psychology construct (Britt et al., 2007). Therefore, it reduces the danger of suicide. From the perspective of the interpersonal-psychological theory of suicide, morale may play a protective role in mental health. Thus, it is inversely associated with suicidal ideation. Thus, we proposed the following hypothesis:

Hypothesis 1. Army morale is negatively correlated with suicidal ideation.

### Self-Control as a Mediator

Some researchers consider self-control to be an aspect of inhibitory control and have defined it as a cognitive process to regulate emotions, thoughts, and behaviors in the face of temptations and impulses (Diamond, 2013). Conceptually, self-control is highly correlated with an individual’s willpower. The strength model of self-control suggests that the self-control process functions like muscles, and the activation of self-control consumes energy/strength/resources (Baumeister et al., 2007). Both the limited-resource theory and the non-limited-resource theory state that the energy/strength/resource behind self-control is willpower (Job et al., 2010). Willpower is considered to be a characteristic that military personnel should possess (Magnussen and Boe, 2021). Troops with high morale were willing to fight longer and harder against greater odds than those exhausted physically and mentally (Dracopoli, 2014). Based on previous research, morale may be connected with self-control through willpower. Owing to an interaction of personal and interpersonal psychological constructs, morale could elicit willpower (Chen, 2013). Thus, it may also promote self-control.

Self-control may also be related to suicide. As stated above, it is central to regulating emotions, thoughts, and behaviors in the face of temptations and impulses. In other words, it suppresses inappropriate emotions, desires, and actions (Casey and Caudle, 2013). Casey and Caudle suggested that some preventable forms of death (accidental fatalities, suicide, and homicide) among teenagers occur primarily because adolescents put themselves in harm’s way, in part due to impaired self-control (Casey and Caudle, 2013). Xie et al. (2020) reported that self-control mediated the effect of childhood abuse on suicidal ideation; childhood abuse diminished individuals′ self-control, which led to higher levels of suicidal ideation. Researchers believe that relatively lower levels of self-control are associated with accumulative stress (Hamilton et al., 2014). This stress is acknowledged to be a leading factor of suicidal ideation (Wilburn and Smith, 2005).

In conclusion, we proposed the following hypothesis:

Hypothesis 2. Self-control may mediate the relationship between army morale and suicidal ideation.

### Interdependent Self-Construal as a Moderator

Self-construal may be inversely related to suicidal ideation. The concept of self-construal was first demonstrated by Markus and Kitayama (1991) to explain different patterns in how the “self” was constructed. Individuals with independent self-construal prioritized the self over the group: they sought independence and autonomy and preferred separateness from others. In contrast, individuals with interdependent self-construal prioritized the group over the self, seeking to maintain harmony and fit into the group. Surprisingly, previous studies have reported that self-construal may be associated with mental distress (Liu and Goto, 2007), which is a leading risk factor for suicidal ideation (Feng et al., 2015). Individuals endorsing higher levels of interdependence place a higher value on social relations than individuals who favor independence. This valuing could foster more positive interpersonal interactions, with benefits such as social support. This interpersonal advantage promotes good mental health (Suro and Weisman De Mamani, 2013). Mentally healthy individuals are less likely to commit suicide. This path may delineate how self-construal negatively relates to suicidal ideation.

Meanwhile, self-construal may interact with army morale related to suicidal ideation. As previously mentioned, low interdependent (independent) individuals lack initiative in social relations. They may experience a lack of positive interpersonal interactions. According to Suro and Weisman De Mamani (2013), such a deficit may lead to relatively low levels of mental health and a greater risk of suicidal ideation. Nevertheless, army morale may play a compensatory role. Previous studies have indicated that better morale in the military context may actively promote positive interpersonal support from peers and leaders, promoting mental health (Jones et al., 2012). Morale’s effect may compensate for interpersonal insufficiency among less interdependent individuals and help them cope more effectively with suicidal ideation. Based on previous studies, we proposed the following hypothesis:

Hypothesis 3. Interdependent self-construal may interact with army morale against suicidal ideation.

### The Current Study

**FIGURE 1:**
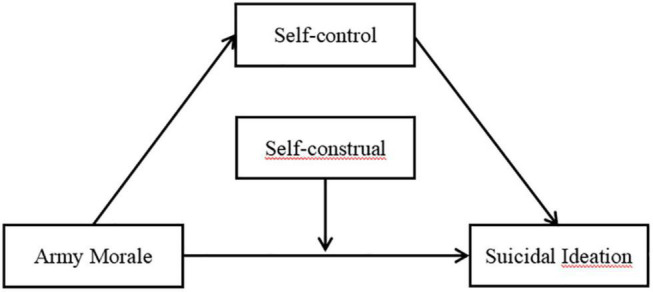
Theoretical moderated mediation model.

From the perspective of interpersonal-psychological theory, the current study examined the relationship between army morale and suicidal ideation. It hypothesized that this relationship is mediated by self-control and moderated by interdependent self-construal. Following Hayes′ suggestion (Hayes, 2013), a moderated mediation model was constructed and used in this study. The proposed moderated mediation model is illustrated in Figure 1.

## Materials and Methods

### Participants

Using the cluster sampling method, the current study recruited 1124 male navy cadets from a military university in China in December 2020. The participants were between 18 and 33 years of age (*M*_age_ = 21.62, *SD*_age_ = 1.56). All participants provided written informed consent and were instructed to complete a series of questionnaires in a random order, which was a paper-and-pencil task. All questionnaires were completed anonymously to ensure the validity of the study. After the participants completed the questionnaires, they were thanked and given notebooks as a gift. All the steps performed in this study were in accordance with the Declaration of Helsinki.

### Measurements

**Army morale** was measured using the Army Morale Scale (Zhu, 2019). Participants were asked to respond to 25 items using a 5-point scale ranging from 1 (strongly disagree) to 5 (strongly agree). Higher scores represented higher morale levels. For example, one item from the scale was: All members are proud to be a part of the team. This scale showed adequate internal consistency (full-scale Cronbach’s α = 0.93).

**Self-construal** Scale was developed by Singelis (1994), revised and translated by Pan and Lv (2013). The interdependent self-construal subscale was adopted to measure cadets’ self-construal. Participants were asked to respond to each item on a 7-point Likert scale ranging from 1 (strongly disagree) to 7 (strongly agree), with higher scores representing more interdependency. The subscale consisted of 10 items; one sample item was: It is important for me to maintain harmony within my group. Cronbach’s α for the Self-construal Scale in this study was 0.83.

**Self-control** levels of the cadets were measured using the Self-control Scale (Tangney et al., 2004). This scale is revised and translated by Tao et al. (2021). This scale consists of 36 items assessed on a scale ranging from 1 (not at all) to 5 (very much), with higher scores representing higher self-control levels. One item example was: I do many things in the spur of the moment. The Self-control Scale showed adequate internal consistency in this study (full-scale Cronbach’s α = 0.90).

**Suicidal ideation** of the cadets was measured using the Self-rating Idea of Suicide Scale (SIOSS) adopted from the study by Xiong et al. (2017). The SIOSS consists of 26 items answered with “yes” or “no” (0 = yes, 1 = no), with higher scores representing higher levels of suicidal ideation. An example item from this scale was “I often feel pessimistic and disappointed”. The SIOSS consists of four dimensions; the sum of the first three dimensions (sleep, desperation, and optimism) was used to assess individuals′ suicidal ideation. The last dimension (conceal) was set to detect if the participants concealed answers while completing the scale. Scores higher than 4 had to be excluded from further analysis. The SIOSS showed adequate internal consistency for the study and the overall Cronbach’s α was 0.88.

### Data Analysis

Missing data were handled with mean imputation (Little and Rubin, 2002). Using the Statistical Package for the Social Sciences (SPSS) version 25, the analysis was divided into the following steps:

1. Pearson’s correlation coefficients were calculated to reveal potential relationships between variables. Among the variables, age was included as a covariate. Sex was not included in the analysis because all participants were male cadets.
2. The mediating effect of self-control between morale and suicidal ideation was tested using Model 4 from SPSS Marco PROCESS (Hayes, 2013). All raw data were standardized before testing the mediation effect to obtain the standardized regression coefficients.
3. Based on the previous steps, Model 5 from PROCESS was adopted to test the full moderated mediation model, including the mediating effect of self-control and the moderating effect of self-construal between morale and suicidal ideation. The bootstrapping method was applied to test for the significance of the effects to obtain robust standard errors for parameter estimation. This method produced 95% bias-corrected confidence intervals for these effects from 1,000 resamples of the data. Confidence intervals that do not contain zero indicate significant effects.

## Results

### Common Method Bias

To control for common method bias, reverse-coded items were utilized and measurements were collected anonymously. A Harman single-factor method was also adopted during the data analysis to search and control for common method bias. The unrotated results of an exploratory factor analysis obtained a total of 11 factors. The interpretation rate of the first factor was 26.23%, which is less than the 40% threshold, beyond which results are considered unreliable. The results indicated that no serious common method bias existed in the study.

### Bivariate Analyses

Pearson correlation coefficients between the variables were calculated and are presented in Table 1. After controlling for age as a covariate, army morale was significantly correlated with the other variables, including self-control (*r* = 0.51, *p* < 0.001, 95% CI [0.46, 0.55]), self-construal (*r* = 0.44, *p* < 0.001, 95% CI [0.39, 0.49]), and suicidal ideation (*r* = –0.29, *p* < 0.001, 95% CI [–0.34, –0.23]). Self-control was significantly correlated with self-construal (*r* = 0.37, *p* < 0.001, 95% CI [0.32, 0.42]) and suicidal ideation (*r* = –0.36, *p* < 0.001, 95% CI [–0.41, –0.31]). Self-construal was negatively correlated with suicidal ideation (*r* = –0.19, *p* < 0.001, 95% CI [–0.25, –0.13].

**TABLE 1:** Descriptive statistics and inter-correlations between variables.

Variables	*M*	*SD*	1	2	3	4	5
Army morale	106.21	18.22	1				
Self-control	3.90	0.60	0.51***	1			
Interdependent Self-construal	57.45	7.96	0.44***	0.37***	1		
Suicidal ideation	2.80	3.42	–0.29***	–0.36***	–0.19***	1	
Age	21.62	1.53	–0.03	–0.05	0.03	–0.01	1

M = mean; SD = standard deviation; ***p < 0.001.

### Testing the Mediation Model

**FIGURE 2:**
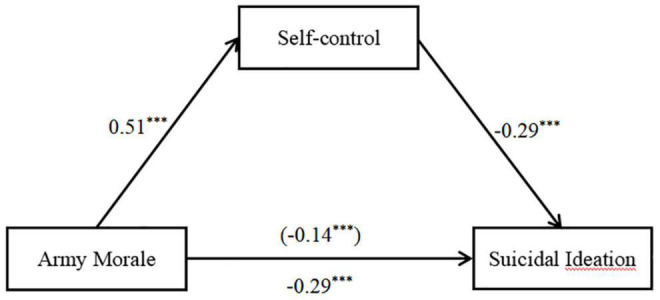
Test of the mediating effect of self-control in the association between army morale and suicidal ideation. The numbers are standardized regression coefficients. Paths between controlled variable (age) and each of the variables in the model are not displayed. ***p < 0.001.

Using Model 4 from SPSS PROCESS, the mediating role of self-control in the relationship between army morale and suicidal ideation was examined. With age controlled as a covariate, the results indicated that army morale negatively predicted suicidal ideation (β = –0.29, *t* = –10.05, *p* < 0.001). Moreover, this predictive effect remained significant after adding the mediation variable of self-control to the model (β = –0.14, *t* = –4.40, *p* < 0.001). The negative predictive effect of self-control on suicidal ideation was also significant (β = –0.29, *t* = –8.97, *p* < 0.001). Moreover, army morale had a significant predictive effect on self-control (β = 0.51, *t* = 19.778, *p* < 0.001). As hypothesized, self-control partially mediated the significant predictive effect of army morale on suicidal ideation. Specifically, the direct effect size was –0.14 (95% CI [–0.20, –0.08]) and the indirect effect was —0.15 (95% CI [–0.19, –0.11]), accounting for 51.00% of the total effect (see Figure 2 and Table 2).

**TABLE 2:** Testing the mediation effect of self-control.

Predictors (IV)	Model 1 (DV: suicidal ideation)		Model 2 (DV: self-control)		Model 3 (DV: suicidal ideation)	
	β	*t*	*B*	*t*	β	*t*
Army morale	–0.29	–10.05***	0.51	19.77***	–0.14	–4.40***
Self-control					–0.29	–8.97***
Age	–0.02	–0.63	–0.03	–1.22	–0.03	–0.98
R2	0.08		0.26		0.14	
F	50.61***		196.99***		62.93***	

Variables in the models are standardized; IV, Independent Variable; DV, Dependent Variable; ***p < 0.001.

### Testing the Moderated Mediation Model

**FIGURE 3:**
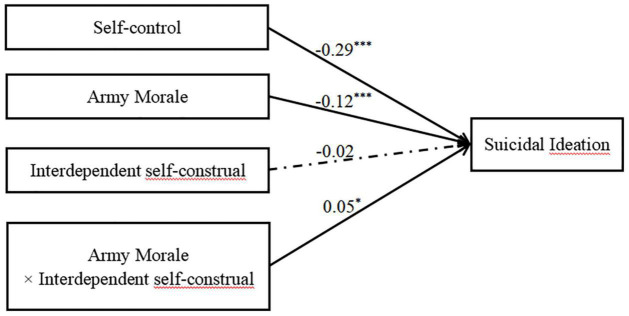
Test of the moderating effect of interdependent self-construal on the association between army morale and suicidal ideation. The numbers are standardized regression coefficients. The dotted line indicates a non-significant relationship. Paths between controlled variables (age) and each of the variables in the models are not displayed. *p < 0.05, ***p < 0.001.

**FIGURE 4:**
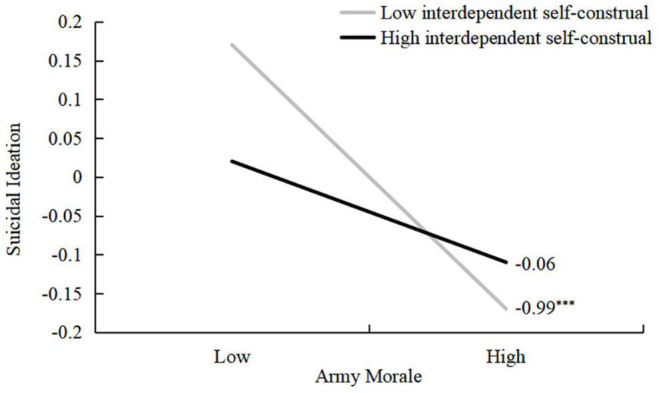
The moderating effect of interdependent self-construal on the relation between army morale and suicide ideation. Variables in the models are standardized. Army morale and interdependent self-construal are graphed for two levels: 1 standard deviation above the mean is high level, and 1 standard deviation below the mean is low level. ***p < 0.001.

Using Model 5 from SPSS PROCESS, the mediating role of self-control and the moderating role of self-construal in the relationship between army morale and suicidal ideation were examined. As shown in Figure 3 and Table 3 and, with age controlled as a covariate, the results of Model 1 in the moderated mediation analysis revealed that army morale had a significant predictive effect on self-control (β = 0.51, *t* = 19.77, *p* < 0.001). Furthermore, the results of Model 2 in the moderated mediation analysis revealed that the predictive effects of morale (β = –0.12, *t* = –3.42, *p* < 0.001) and self-control (β = –0.29, *t* = –8.73, *p* < 0.001) on suicidal ideation were significant. The predictive effect of interdependent self-construal on suicidal ideation was not significant (β = –0.02, *t* = –0.70, *p* = 0.48). However, there was a significant interaction between army morale and interdependent self-construal regarding suicidal ideation (β = 0.05, *t* = 2.10, *p* < 0.05). As shown in Figure 4, the simple slope analysis revealed that army morale negatively predicted suicidal ideation among cadets with lower levels of interdependent self-construal, *b*_simple_ = –0.99, *t* = –4.44, *p* < 0.001, 95% CI [–0.25, –0.10]. However, this predictive direct effect was not significant among cadets with high levels of interdependent self-construal, *b*_simple_ = –0.06, *t* = –1.37, *p* = 0.17, 95% CI [–0.16, 0.03].

**TABLE 3:** Testing the moderated mediation model.

Predictors (IV)	Model 1 (DV: self-control)		Model 2 (DV: suicidal ideation)	
	β	*t*	β	*t*
Army morale	0.51	19.77***	–0.12	–3.42***
Self-control			–0.29	–8.73***
Interdependent self-construal			–0.02	–0.70
Army morale × Interdependent self-construal			0.05	2.10*
Age	–0.03	–1.22	–0.02	–0.83
R2	0.26		0.14	
F	196.99***		38.89***	

Variables in the models are standardized; IV, Independent Variable; DV = Dependent Variable; *p < 0.05, ***p < 0.001.

## Discussion

The current study suggested that army morale may be negatively associated with suicidal ideation. Furthermore, this predictive effect was mediated by self-control and moderated by interdependent self-construal. Specifically, stronger morale led to better self-control and, in turn, reduced suicidal ideation. The moderated mediation analysis suggested that self-construal also interacted with army morale regarding suicidal ideation: morale negatively predicted suicidal ideation among cadets with lower levels of interdependent self-construal. However, this predictive effect was not significant among cadets with higher levels of interdependent self-construal.

Consistent with the results of previous studies, the current study demonstrated that army morale was a strong protective factor against suicidal ideation. Under the premise that military units face a higher risk of suicide than the general population (Hoyt and Duffy, 2015), the current study is the first to reveal that army morale could negatively predict suicidal ideation in this population.

The current study also examined the mediation effect of self-control between army morale and suicidal ideation. Previous studies, discussed in the Introduction, support the current study’s result: self-control partially mediated the predictive effect of morale on suicidal ideation. These findings suggested that army morale could not only predict suicidal ideation, but also predict self-control (Zelenkov, 2001; Magnussen and Boe, 2021), thus reducing suicidal ideation by suppressing inappropriate emotions, desires, and actions (Casey and Caudle, 2013). Example of damaging factors include the challenges of cadets’ daily training, tension, combat stress, and isolation from family members and friends.

The moderated mediation analysis results suggested that the negative predictive effect of morale on suicidal ideation was moderated by self-construal. Cadets’ morale significantly predicted suicidal ideation when interdependent self-construal was low. However, this effect was not significant among cadets with high levels of interdependent self-construal. Those with a more independent self-construal tendency prioritize the self over the group and prefer independence, autonomy, and separateness from others. In contrast, those with a more interdependent self-construal tendency regard the group more than the self and seek to maintain harmony and fit into the group (Markus and Kitayama, 1991). In the current study, a greater interdependent self-construal failed to predict cadets’ suicidal ideation individually, but the interaction between army morale and interdependent self-construal was significant. Joiner et al. (2009) suggested that a lack of social belonging and the existence of social alienation are key to suicidal ideation. Thus, morale and interdependent self-construal may be compensatory sources of social function to prevent suicidal ideation, but army morale is a relatively stronger predictor of suicidal ideation compared to interdependent self-construal.

Therefore, it can be understood that cadets with low interdependence are more sensitive to social isolation and alienation. The lack of social interaction motives may increase suicidal ideation. However, morale may buffer such risk and protect low interdependent cadets from suicidal ideation in the military context.

The study’s findings offer potential applications for military units seeking to prevent suicidal ideation and risk of suicide among soldiers, including suggestions for building stronger morale in military units. Specifically, low interdependent soldiers may need special attention as it may be more sensitive to the social isolation and alienation common among soldiers and cadets. As existent studies have suggested, the expectations from soldiers and their training are becoming increasingly severe. Soldiers now have longer and more frequent deployments, which leads to less time with family members, friends, and communities (Kelly et al., 2014) and hence increases the risk of suicidal ideation. For example, Whitesell and Owens (2012) suggested that supportive leadership behavior may promote morale. Therefore, it reduces the amount of stress, a risk factor for suicidal ideation. This study indicates that leaders in the military unit should be cautious in formulating their leadership style. Moreover, some structured programs, such as the Army Morale, Welfare, and Recreation (MWR) programs, may prevent low military morale and further improve army life quality (Marshall-Mies et al., 2011).

### Limitations and Further Research

The current study examined the relationship between army morale and suicidal ideation. These conclusions advance the understanding of how army morale negatively predict suicidal ideation. However, this study has several limitations, which can be addressed by future researchers. First, it was designed based on questionnaire measures, which made it impossible to draw causal conclusions. Future studies should focus on designing experiments, such as behavioral manipulations, that examine these conclusions in more depth. Second, this study was based on only one group of cadets, and only males were sampled. People from different geographic locations and diverse genders and ethnicities must be sampled to validate and generalize the current conclusions. Finally, although multiple existent studies concluded that self-construal predicted suicidal ideation were reviewed, self-construal failed to predict suicidal ideation in the current study. Thus, the relationship between these two aspects requires further examination.

Moreover, the current study may provide some theoretical and practical implications. The findings may suggest that building stronger morale in military units could prevent suicidal ideation. Previously, the United States army launched the Comprehensive Soldier Fitness (CSF) program to enhance soldiers′ mental health (Casey, 2011). Similar programs should be developed to assess and diagnose potential soldiers and cadets, especially individuals with less interdependent self-construal and bring them into the purview of training programs. For example, along with army units′ daily training, some team competitions may be incorporated into such training programs to enhance army morale.

## Data Availability Statement

The datasets presented in this study can be found in online repositories. The names of the repository/repositories and accession number(s) can be found below: https://osf.io/cdps5/?view_only=fe879bba0b474f7594052c43597abc1b.

## Ethics Statement

The studies involving human participants were reviewed and approved by Institutional Review Board Form of Psychology, Qingdao University. The patients/participants provided their written informed consent to participate in this study.

## Author Contributions

OC designed the study and wrote the manuscript. RL collected and analyzed the data. XZ collected the data and proofread the manuscript. All authors contributed to the article and approved the submitted version.

## Conflict of Interest

The authors declare that the research was conducted in the absence of any commercial or financial relationships that could be construed as a potential conflict of interest.

## Publisher’s Note

All claims expressed in this article are solely those of the authors and do not necessarily represent those of their affiliated organizations, or those of the publisher, the editors and the reviewers. Any product that may be evaluated in this article, or claim that may be made by its manufacturer, is not guaranteed or endorsed by the publisher.

## Footnotes

1
https://www.who.int/publications/i/item/9789240026643

2
https://www.dspo.mil/qsr/

## References

[B1] Akhtar T. Shah R. U. Ghazi S. R. Khalil Y. K. (2016). Morale as Predictor of Secondary School Teachers’ Performance: A Study of the Schools of Khyber Pakhtunkhwa. *Dialogue* 11 388–400.

[B2] Baumeister R. F. Vohs K. D. Tice D. M. (2007). The strength model of self-control. *Curr. Direct. Psychol. Sci.* 16 351–355. 10.1111/j.1467-8721.2007.00534.x

[B3] Bowers C. A. Baker D. P. Salas E. (1994). Measuring the importance of teamwork: the reliability and validity of job/task analysis indices for team-training design. *Military Psychol.* 6 206–214. 10.1207/s15327876mp0604_1

[B4] Britt T. W. Dickinson J. M. Moore D. Castro C. A. Adler A. B. (2007). Correlates and consequences of morale versus depression under stressful conditions. *J. Occupat. Health Psychol.* 12 34–47. 10.1037/1076-8998.12.1.34 17257065

[B5] Bush N. E. Reger M. A. Luxton D. D. Skopp N. A. Kinn J. Smolenski D. (2013). Suicides and suicide attempts in the US military, 2008–2010. *Suicide Life Threat. Behav.* 43 262–273. 10.1111/sltb.12012 23330611

[B6] Casey B. J. Caudle K. (2013). The teenage brain: self-control. *Curr. Direct. Psychol. Sci.* 22 82–87. 10.1177/0963721413480170 25284961 PMC4182916

[B7] Casey G. W. Jr. (2011). Comprehensive soldier fitness: a vision for psychological resilience in the US Army. *Am. Psychol.* 66 1–3. 10.1037/a0021930 21219041

[B8] Chen X. (2013). *The Research on Relationships among Psychological Capital, Job Burnout and Morale of Armed police soldiers.* [Ph.D.thesis]. China: Hunan Normal University.

[B9] Chinyere M. N. Iheanaetu B. I. Gladys C. E. (2020). Teachers’ Participation in Students’ and Staff’s Discipline in secondary schools as determinants of teachers’ Morale in Imo State. *Int. J. Orange Technol.* 2 17–23. 10.31149/ijot.v2i8.801

[B10] Diamond A. (2013). Executive functions. *Ann. Rev. Psychol.* 64 135–168. 10.1146/annurev-psych-113011-143750 23020641 PMC4084861

[B11] Dracopoli M. Z. (2014). A New Officer for a New Army: The Leadership of Major Hugh JC Peirs in the Great War. *Gettysburg Hist. J.* 13:6.

[B12] Feng J. Li S. Chen H. (2015). Impacts of stress, self-efficacy, and optimism on suicide ideation among rehabilitation patients with acute pesticide poisoning. *PLoS One* 10:e0118011. 10.1371/journal.pone.0118011 25679994 PMC4332490

[B13] Fennell J. (2014). In search of the ‘X’factor: morale and the study of strategy. *J. Strategic Stud.* 37 799–828. 10.1080/01402390.2013.846856

[B14] Hamilton K. R. Sinha R. Potenza M. N. (2014). Self-reported impulsivity, but not behavioral approach or inhibition, mediates the relationship between stress and self-control. *Add. Behav.* 39 1557–1564. 10.1016/j.addbeh.2014.01.003 24508183 PMC4222178

[B15] Hayes A. F. (2013). *Introduction to Mediation, Moderation, and Conditional Process Analysis: A Regression-Based Approach.* New York, NY: Guilford Press.

[B16] Hoyt T. Duffy V. (2015). Implementing firearms restriction for preventing US Army suicide. *Milit. Psychol.* 27 384–390. 10.1037/mil0000093

[B17] Jiang G. (2015). Suicidal behaviors: risk factor, psychological theory and future research. *Adv. Psychol. Sci.* 8 1437–1452. 10.3724/SP.J.1042.2015.01437

[B18] Jin S. Zhang J. (1998). The effects of physical and psychological well-being on suicidal ideation. *J. Clin. Psychol.* 54 401–413. 10.1002/(sici)1097-4679(199806)54:4<401::aid-jclp2<3.0.co;2-q 9623745

[B19] Job V. Dweck C. S. Walton G. M. (2010). Ego depletion—Is it all in your head? Implicit theories about willpower affect self-regulation. *Psychol. Sci.* 21 1686–1693. 10.1177/0956797610384745 20876879

[B20] Joiner T. E. Jr. Van Orden K. A. Witte T. K. Selby E. A. Ribeiro J. D. Lewis R. (2009). Main predictions of the interpersonal–psychological theory of suicidal behavior: empirical tests in two samples of young adults. *J. Abnorm. Psychol.* 118:634. 10.1037/a0016500 19685959 PMC2846517

[B21] Jones N. Seddon R. Fear N. T. McAllister P. Wessely S. Greenberg N. (2012). Leadership, Cohesion, Morale, and the Mental Health of UK Armed Forces in Afghanistan. *Psychiatry* 75 49–59. 10.1521/psyc.2012.75.1.49 22397541

[B22] Kelly D. R. Matthews M. D. Bartone P. T. (2014). Grit and hardiness as predictors of performance among West Point cadets. *Milit. Psychol.* 26 327–342. 10.1037/mil0000050

[B23] Leighton A. H. (1949). *Human Relations in a Changing World: Observations on the use of the Social Sciences.* New York, NY: E P Dutton and Company. 10.1037/13243-000

[B24] Li C. (2006). Review of morale research. *China J. Health Psychol.* 04 475–477. 10.13342/j.cnki.cjhp.2006.04.053

[B25] Lin L. Wang C. Juanchan, Mo, Yang Y. Huisheng (2018). The Relationship between Impulsivity Traits and Suicide Ideation: A Moderate Mediation Model. *Psychol. Dev. Educ.* 34 369–376. 10.16187/j.cnki.issn1001-4918.2018.03.14

[B26] Little R. J. A. Rubin D. B. (2002). *Statistical Analysis with Missing Data*, Second Edn. Hoboken, NJ: John Wiley & Sons.

[B27] Liu F. F. Goto S. G. (2007). Self-construal, mental distress, and family relations: a mediated moderation analysis with Asian American adolescents. *Cult. Diversity Ethnic Minor. Psychol.* 13 134–142. 10.1037/1099-9809.13.2.134 17500602

[B28] Liu Y. X. Huang W. Q. Zhu W. E. (2012). Suicidal ideation to satisfaction with life and happiness in college freshmen. *Chin. Ment. Health J.* 26 235–238.

[B29] Magnussen L. I. Boe O. (2021). Machine, Machine! Stories about How Border Breaking Experiences from a Combat Fatigue Course Relates to the Development of Willpower and the Educational Concept of Bildung. *Military Behav. Health* 9 101–109. 10.1080/21635781.2020.1742825

[B30] Markus H. R. Kitayama S. (1991). Culture and the self: implications for cognition, emotion, and motivation. *Psychol. Rev.* 98 224–253. 10.1037/0033-295X.98.2.224

[B31] Marshall-Mies J. Westhuis D. Fafara R. (2011). US Army morale, welfare and recreation (MWR) programmes: links to readiness and retention. *Res. Militaris* 3 1–22. 10.21236/ada554038

[B32] Martin J. Ghahramanlou-Holloway M. Lou K. Tucciarone P. (2009). A comparative review of US military and civilian suicide behavior: implications for OEF/OIF suicide prevention efforts. *J. Mental Health Counsel.* 31 101–118. 10.17744/mehc.31.2.a6338384r2770383

[B33] Olivetta E. (2017). “Leadership, Morale and Cohesion: What should be changed?” in *Leadership in Extreme Situations*, eds Holenweger M. Jager M. Kernic F. (Cham: Springer), 75–92. 10.1007/978-3-319-55059-6_5

[B34] Pan L. Lv W. (2013). Application and revision of self-construal scale among working adults. *China J. Health Psychol.* 21 710–712. 10.13342/j.cnki.cjhp.2013.05.011

[B35] Singelis T. M. (1994). The measurement of independent and interdependent self-construals. *Personal. Soc. Psychol. Bull.* 20 580–591. 10.1177/0146167294205014

[B36] Sipos M. L. Wood M. D. Riviere L. A. Adler A. B. (2017). Behavioral Health Adjustment in Reserve Component Soldiers During a Noncombat Deployment to Africa. *Military Psychol.* 26 409–421. 10.1037/mil0000058

[B37] Suro G. Weisman De Mamani A. G. (2013). Burden, Interdependence, Ethnicity, and Mental Health in Caregivers of Patients with Schizophrenia. *Fam. Process* 52 299–311. 10.1111/famp.12002 23763688

[B38] Tangney J. P. Baumeister R. F. Boone A. L. (2004). High self-control predicts good adjustment, less pathology, better grades, and interpersonal success. *J. Personal.* 72 271–324. 10.1111/j.0022-3506.2004.00263.x 15016066

[B39] Tao L. Limei C. Lixia Q. Shuiyuan X. (2021). Reliability and Validity of Chinese Version of Brief Self-Control Scale. *Chin. J. Clin. Psychol.* 1 83–86. 10.16128/j.cnki.1005-3611.2021.01.017

[B40] Whitesell A. A. Owens G. P. (2012). The impact of patriotism, morale, and unit cohesion on mental health in Veterans of Iraq and Afghanistan. *Traumatology* 18 1–7. 10.1177/1534765610395625

[B41] Wilburn V. R. Smith D. E. (2005). Stress, self-esteem, and suicidal ideation in late adolescents. *Adolescence* 40 33–45. 15861616

[B42] Xie Q. Du Y. Liu N. Bo Y. (2020). Childhood Abuse Affects Aggression and Suicidal Ideation: Mediating Effect of Self-Control. *Chin. J. Clin. Psychol.* 2 387–390. 10.16128/j.cnki.1005-3611.2020.02.035

[B43] Xiong L. Guo Y. Yu L. Tang N. Zhang S. Ma Y. (2017). Relationship between Parenting Styles, Suicidal Ideation and Suicidal Attitude of College Students. *China J. Health Psychol.* 02 219–222. 10.13342/j.cnki.cjhp.2017.02.017

[B44] Zelenkov M. Y. (2001). Morale training in foreign armies. *Military Thought* 10:76.

[B45] Zhu H. T. (2019). Charismatic Leadership and Military Team Performance: interplay between Military and Political Commanders and Mediating Role of Morale. *J. Psychol. Sci.* 1217. 10.16719/j.cnki.1671-6981.20190528

